# Perspectives of gene editing for cattle farming in tropical and subtropical regions

**DOI:** 10.1590/1984-3143-AR2022-0108

**Published:** 2023-02-13

**Authors:** Luiz Sergio Almeida Camargo, Naiara Zoccal Saraiva, Clara Slade Oliveira, Allie Carmickle, Diana Rangel Lemos, Luiz Gustavo Bruno Siqueira, Anna Carolina Denicol

**Affiliations:** 1 Embrapa Gado de Leite, Juiz de Fora, MG, Brasil; 2 Department of Animal Science, University of California Davis, Davis, CA, USA; 3 Universidade Federal de Viçosa, Viçosa, MG, Brasil

**Keywords:** genome editing, bovine, livestock, heat stress, CRISPR

## Abstract

Cattle productivity in tropical and subtropical regions can be severely affected by the environment. Reproductive performance, milk and meat production are compromised by the heat stress imposed by the elevated temperature and humidity. The resulting low productivity contributes to reduce the farmer’s income and to increase the methane emissions per unit of animal protein produced and the pressure on land usage. The introduction of highly productive European cattle breeds as well as crossbreeding with local breeds have been adopted as strategies to increase productivity but the positive effects have been limited by the low adaptation of European animals to hot climates and by the reduction of the heterosis effect in the following generations. Gene editing tools allow precise modifications in the animal genome and can be an ally to the cattle industry in tropical and subtropical regions. Alleles associated with production or heat tolerance can be shifted between breeds without the need of crossbreeding. Alongside assisted reproductive biotechnologies and genome selection, gene editing can accelerate the genetic gain of indigenous breeds such as zebu cattle. This review focuses on some of the potential applications of gene editing for cattle farming in tropical and subtropical regions, bringing aspects related to heat stress, milk yield, bull reproduction and methane emissions.

## Introduction

Tropical and subtropical regions are home of about 40% of the world’s human population and where nations with the highest growth rates and poorest populations are located (United Nations Department of Economic and Social Affairs Population Division, 2022). These regions also contain more than 80% of the cattle population (Cooke et al., 2020) and, therefore, have great potential to contribute to fulfill the global demand on animal-source food for a constantly growing population. However, tropical and subtropical environments have been challenging for livestock production. The high temperature and humidity found in several of those regions have a negative impact on animal physiology, altering metabolic and hormonal status (Santos et al., 2021), resulting in low fertility and suboptimal milk and meat production (Summer et al., 2018). The outputs of large numbers of less productive cattle in these regions are low farmer income, high methane emission per unit of milk or meat produced (Oosting et al., 2014) and pressure on land usage (DeFries and Rosenzweig, 2010; Weindl et al., 2017).

In an attempt to improve cattle productivity, breeds from European origin have been introduced in Africa, Asia and Latin America. However, the introduction of breeds that were proved successful in developed nations located in regions of temperate climate usually results in lower efficiency because of the heat stress, low pasture quality (Manteca and Smith, 1994) and parasites (Shyma et al., 2015) found in the tropics. There are two strategies to reduce the effect of heat stress on productivity: to increase the heat tolerance of exotic breeds and/or to increase the productivity of local breeds already adapted to tropical and subtropical environments, such as zebu breeds. Both strategies can be achieved by genetic improvement programs. Local breeds can also be crossed with exotic non-adapted breeds to take advantage of heterosis (Syrstad, 1996, 1989; Miranda and Freitas, 2009). However, these approaches require several generations to change a desirable trait in a population, which takes decades because of the long generation interval in cattle (Jonas and Koning, 2015). In addition, the effect of heterosis is reduced in the following generations (Syrstad, 1989). Finally, although introgression by crossbreeding can transfer genes or alleles associated to favorable traits in a determined breed, it can also transfer alleles of non-desired traits that may further compromise animal fertility and performance.

Gene (or genome) editing tools have been developed and improved in the last two decades. These tools allow the precise introduction of mutations in a given gene (Gaj et al., 2013), for what they are referred to as precision breeding technologies. The Clustered Regularly Interspaced Short Palindromic Repeat (CRISPR) - associated nuclease 9 (CRISPR/Cas9 system) technology is among the most efficient, easiest to use and lowest cost gene editing methods (Kim and Kim, 2014; Zhao et al., 2021; Zhu, 2022). In the CRISPR technology, a small guide RNA (sgRNA) leads a nuclease (Cas9, for example) to a specific location in the genome to create a double-stranded break (DSB) in the DNA (Doudna and Charpentier, 2014). The sgRNA is designed to align to a specific target sequence in the DNA, reducing the chances of targeting undesired sequences (off-targets). Following the action of the nuclease, the repair of the DNA cleavage occurs mainly by non-homologous end joining (NHEJ) of the broken ends. In this process, some nucleotides can be inserted or deleted (indels) and create mutations in the target gene. If it is in frameshift, the mutations can disrupt the gene expression and, consequently, eliminate the production of the encoded protein, or eventually it can also create a stop-codon. This strategy can be useful to knock out the expression of a specific protein or to generate a truncated protein in a given organism. The cell can also repair the DSB by the homology-directed repair (HDR) mechanism. In this case, an oligodeoxynucleotide (ODN) donor template homologous to the target region designed with a target mutation is used. This ODN donor template containing the mutation is then inserted by homology into the cell genome during the DSB repair. However, HDR is much less frequent than the NHEJ mechanism (Liu et al., 2019) and is restricted to the G2 and S phases of the cell cycle (Symington and Gautier, 2011; Takata et al., 1998), making gene editing by HDR less common than NHEJ.

The applications of the CRISPR system in different fields of biology has been shown in several reports (Carroll, 2017; Doudna, 2020; Molla et al., 2021). In cattle, the application of this technology opens the opportunity to accelerate genetic improvement via the faster dissemination of desirable traits. The technology allows alleles associated with desirable traits in a particular breed to be introduced into another breed without crossbreeding, or to increase the frequency of such alleles in a given population (Hickey et al., 2016). Gene editing together with genomic selection has the potential to double the genetic gain after 20 years when multiple edits are performed (Jenko et al., 2015).

Assisted reproductive technologies such as somatic cell nuclear transfer (animal cloning) or in vitro fertilization (IVF) are required to generate gene-edited embryos. When IVF is applied, a large number of gene-edited animals can be produced in one or two generations by commercial in vitro fertilization laboratories. The gene-edited embryos can be biopsied so that the genomic evaluation can be performed in order to select the ones with high estimated genomic values before transferring them into recipients. Thus, gene editing together with assisted reproductive technologies and genomic selection can play a major role in genetic breeding programs by either reducing the generation interval, increasing selection intensity and accuracy, and/or by increasing genetic variation (Mueller and Van Eenennaam, 2022).

In this review we will focus on how cattle farming can benefit from gene editing technologies in the tropical and subtropical regions. This review will cover aspects related to gene editing applications regarding heat stress, milk yield and composition, bull reproduction and methane emissions.

## Gene editing to alleviate the effects of heat stress on European cattle

Several breeds of *Bos taurus* cattle from central and south America such as Senepol, Romosinuano, Criollo Limonero and Carora have been selected for adaptation to tropical conditions. One of the first studies examining the thermotolerance of *B. taurus* cattle from the tropics was performed by Hammond et al. in subtropical Florida to compare the rectal temperature (RT) of Senepol, Angus, Hereford and Brahman cattle during summer (Hammond et al., 1996). The authors found that Senepol and Brahman had similar temperature, which was lower than Hereford and Angus animals. Crossbreeding of Hereford and short hair Senepol revealed that the offspring inherited the short hair phenotype and lower RT typical of Senepol cattle. When investigating this phenomenon further, Olson et al. performed backcross mating with Holstein, Charolais, or Angus to Senepol or Carora crosses and found evidence of a major gene with dominant inheritance responsible for creating the short, sleek hair coat phenotype seen in the tropical breeds (Olson et al., 2003). They reported lower RT in crossbred calves (0.18-0.4 °C) and lactating cows (0.61 °C) that had short hair when compared to normal-haired contemporaries.

In 2014, Littlejohn et al. described a causative mutation in the prolactin receptor gene (*PRLR*) responsible for the short (slick) hair coat phenotype (Littlejohn et al., 2014). A frameshift mutation resulting from a single cystine deletion caused a premature stop codon (p.Leu462*) in the resulting protein. This mutation became known as the SLICK1 allele and, although the SLICK1 has been the best characterized mutation so far, additional variants of the *PRLR* have been reported that result in truncation of the protein at different points and causing the similar slick phenotype observed in criollo-derived *B. taurus* breeds (Porto-Neto et al., 2018; Flórez Murillo et al., 2021). These alleles have been named SLICK2-SLICK6 (Flórez Murillo et al., 2021). Matings between Senepol and Holsteins were performed in Florida and Puerto Rico since the 1980s, and nowadays there are several registered Holstein sires that carry the SLICK1 allele. In Puerto Rico, crosses between Holsteins and other thermotolerant criollo breeds found in the Caribbean were done for many years before the introduction of the Senepol. As a result, Puerto Rican Holsteins are still genotyped as having the SLICK1 allele, but the mutation is most likely to have been introduced via a shared common ancestor between Senepol and the other criollo breeds in Puerto Rico (Hansen, 2020).

The thermotolerance of slick cattle during periods of heat stress have been mostly evaluated in regions of high humidity heat. Lactating slick Holstein cows had lower rectal and vaginal temperatures and respiratory rates during summer compared to non-slick contemporaries (Dikmen et al., 2008, 2014). Slick-haired Criollo Limonero non-pregnant heifers had lower rectal temperature and respiratory rates than normal-haired heifers (Landaeta-Hernández et al., 2021). Pre-weaned Holstein calves and growing heifers carrying the SLICK1 allele also maintained lower rectal temperature when exposed to high-humidity heat during summer (Carmickle et al., 2022). Criollo Limonero cattle slick females had larger sweat glands (more consistent with those of *B. indicus* cattle) compared to wild-type females (Landaeta-Hernández et al., 2011). However, no differences between the number of sweat or sebaceous glands, or hair follicles per square centimeter, thickness of epidermis, or number of blood vessels per square centimeter between genotypes were found. Later studies found that slick Holstein cows had larger cross-sectional sweat gland area and perimeter compared to wild-type cows (Contreras-Correa et al., 2017) and similar to that of Senepol cattle (Muñiz-Cruz et al., 2018).

One of the major expected effects of the slick cattle thermotolerance is a less dramatic drop in milk yield during periods of heat stress as seen during summer months. In an arid region of Venezuela, slick-haired 3/4 Holstein x Carora crossbred cows had greater 305-d milk yield and lower rectal temperature than normal-haired 3/4 Holstein x Carora (Olson et al., 2003). In Florida, USA, the milk yield of Holstein cows carrying the SLICK1 allele dropped on average 1.3 kg/day during the hot season compared to the cool season, whereas non-slick cows dropped on average 3.7 kg/day (Dikmen et al., 2014). In Puerto Rico, slick-haired local Holstein cows had an increased grazing time under sunlight and produced on average 4.27 kg/day more milk than normal-haired cows during summer (Sánchez-Rodríguez and Domenech-Pérez, 2021). Although semen from a few dairy sires with SLICK mutations is available in the North American market, most of them are heterozygous and exhibit a low merit genetic when compared to their normal-haired counterparts. The only SLICK homozygous sire has a negative TPI (UF/IFA Range Cattle Research and Education, 2022).

Considering the positive effects of SLICK alleles on thermotolerance and the predicted effect on milk yield, mutations in the *PRLR* gene are strong candidates for gene editing in order to generate more heat-tolerant European dairy cows for tropical and subtropical regions. SLICK homozygous embryos from high genomic value sires and dams can be produced by introducing any of the SLICK mutations. To introduce one of those specific mutations in the *PRLR* gene, the DSB needs to be repaired by homologous recombination (HDR mechanism). For that, a single strand ODN (ssODN) donor template designed with one of the SLICK mutations can be used. As the HDR is not the usual DSB repair pathway employed by cells, the chances to introduce some of the SLICK mutations are limited. However, as the different mutations of SLICK alleles found in the different breeds are located between BTA20:39099113 and BTA20:39099321 positions (ARS-UCD1.2 genome assembly) (Flórez Murillo et al., 2021), it is likely that any stop-gain mutation introduced in that range will result in similar phenotype. This means that the DSB can be repaired by the HDR mechanism using a ssODN donor template designed with a stop-gain mutation that fits into this range, i.e., not necessarily with the mutations presented in the SLICK alleles. In addition, if the DSB is repaired by the NHEJ, which is the more frequent mechanism of DSB repair, insertions and/or deletions of nucleotides can occur in-between those genome positions and also generate a nonsense mutation. Thus, different gene editing approaches can be used to edit the *PRLR* gene, which would make it easier to generate animals with the SLICK phenotype.

**Figure 1:**
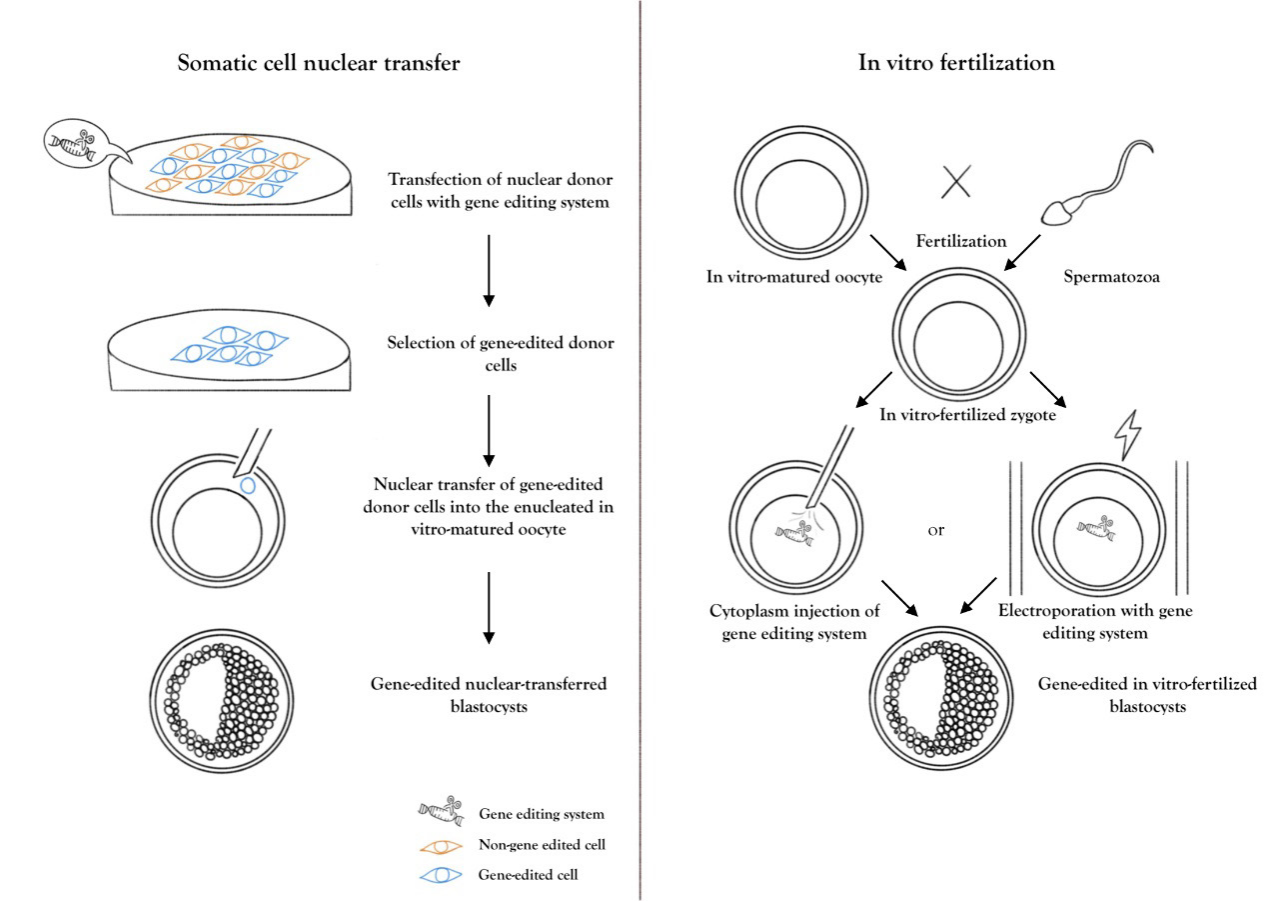
Gene-edited embryos can be produced by either somatic cell nuclear transfer (SCNT) or in vitro fertilization (IVF). While in SCNT the somatic cells are edited by CRISPR systems and in vitro-selected before serving as nuclear donors to generate a cloning embryo, in IVF the matured oocyte or in vitro-fertilized zygote are injected or electroporated with the CRISPR system.

SLICK animals can be generated from embryos produced by nuclear transfer performed with gene-edited somatic or embryonic stem cells (animal cloning), or from in vitro fertilized zygotes injected or electroporated with CRISPR/Cas9 (Figure 1). The animals derived from those embryos can then be used to breed European cattle raised in regions of high temperature and humidity index. The same approach can be used for thermosensitive European beef breeds, such as Angus, so that sires can have higher tolerance to heat stress during the breeding season in the tropics. Few gene-edited SLICK animals have been generated by a commercial company and demonstrated the feasibility of editing this specific gene in cattle.

## Gene editing to improve milk yield and composition in indigenous cattle in tropical and subtropical regions

It has been challenging to maintain efficient dairy production systems in tropical and subtropical regions. Because of heat stress, high productivity European breeds cannot produce milk as they usually do in temperate climates. On the other hand, despite the adaptation to the tropical environment, local indigenous (or adapted) breeds have low productivity, in part due to the lack of well-established genetic improvement programs. Heat stress also affects milk composition. Protein and fat content can be reduced, which may alter the coagulation properties of the milk used to make cheese, affecting cheese yield (Summer et al., 2018).

Gene editing offers the possibility to enhance milk production and to improve milk composition in dairy cattle. Polymorphisms associated with milk yield and composition can be introduced in a given breed without crossbreeding exotic and local breeds. One example is the polymorphism in the growth hormone receptor (*GHR*) gene. One of the actions of the growth hormone (GH) is to stimulate milk and protein production, which can occur indirectly through systemic changes, such as food intake, blood flow and nutrient delivery to the mammary gland (Bauman, 1999) but may also involve direct mechanisms through GHR present in the epithelial cells of the mammary gland (Svennersten-Sjaunja and Olsson, 2005). Some Holstein and Jersey animals have a mutation in exon 8 of the *GHR* gene that results in an amino acid change (phenylalanine>tyrosine). The resulting allele (Y) is associated with higher milk production (Blott et al., 2003; Viitala et al., 2006; Rahmatalla et al., 2011), higher protein percentage (Sun et al., 2009) and lower somatic cell count (Rahmatalla et al., 2011). The Y allele accounted for a variation between 0.7 and 2.9% in milk yield in Dutch and New Zealand Holsteins cows, with a deviation between 67 and 162 kg in the first lactation (Blott et al., 2003) and an increase in milk yield by 320 kg per lactation in German Holstein cows (Rahmatalla et al., 2011). This mutation has not been reported in indigenous cattle so far (Ramesha et al., 2016; El-Nahas, 2018).

The fat content of milk has been reported to decrease during summer or under high temperature and humidity index conditions (i.e., under conditions leading to heat stress) (Liu et al., 2017; Summer et al., 2018), although unsaturated fatty acid indicators can increase (Bohlouli et al., 2021; Penev et al., 2021). Diacylglycerol acyltransferase 1 (DGAT1) is an enzyme that acts on triacylglycerol metabolism (Bhatt-Wessel et al., 2018). The K232A mutation in the *DGAT1* gene is a non-synonymous substitution of lysine to alanine that was found to be associated not only with milk fatty acid content (Grisart et al., 2002; Winter et al., 2002) but also with milk yield in cows (Grisart et al., 2002). The K allele has been associated with higher percentage of C6:0, C8:0, C16:0 and C16:1 fractions, as well as a lower percentage of C14:0, C18:1 and CLA fractions in the milk (Bouwman et al., 2011; Kęsek-Woźniak et al., 2020). The A allele has been reported to contribute to increase the proportion of unsaturated fatty acids (Tăbăran et al., 2015; Bovenhuis et al., 2016), which is considered beneficial to human health (Lee and Park, 2014). It was suggested that the effect of the K232A polymorphism on milk fat synthesis and composition may be caused by differences in the membrane organization or cell structure of epithelial cells in the mammary gland between KK and AA genotype (Lu et al., 2015). The A allele has also been associated with higher milk yield in Holstein cows (Bovenhuis et al., 2015; Grisart et al., 2002), and the estimated effect of AA over the KK genotype on milk yield was 774 kg, 1,042 and 1,028 kg milk for first, second and third lactation, respectively (Bovenhuis et al., 2015). The A allele is presented in frequencies over 50% in Holstein breed (Banos et al., 2008; Näslund et al., 2008; Bobbo et al., 2018). In contrast, the frequency of A allele in zebu cattle was reported to be lower than 5%, as reported for Gir (4%) and Red Sindhi (2.5%) breeds (Lacorte et al., 2006). Low frequency of A allele was also found in African indigenous cattle, as Borgou (23%) and White Fulani (8%) breeds (Houaga et al., 2018). As result, milk from those African breeds exhibited a high percentage of total saturated fatty acids and low C18 unsaturation index.

The stearoyl-CoA desaturase 1 (SCD1) is an enzyme responsible for fatty acid desaturation in the mammary gland and other tissues, playing an important role in lipid metabolism of mammary tissues by introducing a cis double bond at the C-9 position of a wide range of fatty acids (Paton and Ntambi, 2009; Jacobs et al., 2013). The preferred substrate is C18:0 and to a lesser extent C16:0, which are converted to C18:1 cis-9 and C16:1 cis-9, respectively (Ntambi and Miyazaki, 2004). The enzyme SCD1 plays a vital role in maintaining the fluidity of the cell membrane and milk fat. SCD1 is also responsible for the conversion of C18:1 trans-11 to C18:2 cis-9, trans-11 which in turn has been linked to human health benefits (Bhattacharya et al., 2006; Reynolds and Roche, 2010). There is a non-synonymous mutation (A293V) in the *SCD1* gene that results in an alanine to valine substitution in the enzyme and it is associated to changes in milk fatty acid composition in Holstein cows (Mele et al., 2007; Schennink et al., 2008; Kęsek-Woźniak et al., 2020). Milk fat of AA-genotype Holstein cows can have higher content of mono unsaturated fatty acids, as C14:1 *cis-9* and C18:1 *cis*-9 (Mele et al., 2007), and also polyunsaturated fatty acids, as *cis*-9, *trans*-11 conjugated linoleic acid (Schennink et al., 2008), although this later effect is controversial and may be influenced by the diet (Clark et al., 2010). The frequency of the A allele has been reported to be over 50% in Holstein (Kgwatalala et al., 2007; Demeter et al., 2009; Wulandari et al., 2019; Kęsek-Woźniak et al., 2020) and Jersey (Kgwatalala et al., 2007) breeds, while African indigenous breeds such as the White Fulani were found to have a high frequency (>83%) of the V allele, which was associated with a lower C18:1 *cis-9* percentage in milk (Houaga et al., 2018).

As shown above, the alleles found in *GHR* (Y allele), *DGAT1* (A allele) and *SDC1* (A allele) genes are the result of point mutations presented in higher frequency in European dairy breeds compared to indigenous breeds, and are associated to milk yield and fat content. Thus, those point mutations are potential candidates to increase the milk yield and improve milk composition in adapted indigenous breed in tropical and subtropical zones, such as zebu dairy breeds. The introgression of those alleles in the genome of dairy breeds can be performed by gene editing, preserving other racial features of indigenous breeds.

The HDR mechanism is required to insert these point mutations into the genome. For that, the DSB caused by the Cas9 enzyme (or other nucleases) can be repaired using a ssODN donor template designed with the target mutation and homology arms (upstream and downstream of the mutation site). Moreover, the donor template must contain a silent mutation to avoid the re-cut of the repaired DNA by the Cas9. Finally, to increase the chances of successful HDR, the DNA cleavage site needs to be as close as possible from the mutation insertion site (Paquet et al., 2016; Schubert et al., 2021). The problem is that HDR occurs in a frequency usually below 10% (Liu et al., 2019).

Strategies can be employed to increase the chances of HDR over the NHEJ mechanism for DSB repair. Some small molecules can act to inhibit NHEJ while others can be used to stimulate HDR. One of the NHEJ inhibitor molecules is SCR7, which acts by inhibiting the DNA ligase IV enzyme, necessary for double-strand break repair (Ryu et al., 2019). In mice it was possible to obtain 59% HDR (versus 28% NHEJ) in blastocysts when using 1 µM of SCR7 in the cytoplasmic microinjection with CRISPR/Cas9 (Maruyama et al., 2015). In fetal porcine cells, SCR7 increased the HDR rate by 2-3 times (Li et al., 2017); however, no improvements were observed in rabbit zygotes (Song et al., 2016). RS-1 is another small molecule and it is important for catalyzing the repair by homologous recombination by stimulating the function of the Rad51 protein (DNA repair protein). A concentration of 7.5 µM RS-1 in the post-microinjection culture increased the HDR rate in rabbit embryos to 24%, as measured by the knock in proportion, when compared to 4.4% in the control group; a similar difference was also observed in the born animals (Song et al., 2016). In bovine embryos, culturing zygotes with 7.5 µM RS-1 for 24h after microinjection of CRISPR/Cas9 doubled the HDR rate (Lamas‐Toranzo et al., 2020).

## Gene editing to improve bull reproduction in tropical and subtropical regions

The high temperature and humidity index typical of tropical and subtropical climates alters the behavior of breeds from European origin; examples of behavioral changes are a decrease in dry matter intake and the seeking for shade (Mishra, 2021). Moreover, environmental heat stress can affect sperm quality (Morrell, 2020) and reduce bull fertility (Rahman et al., 2018). Thus, heat stress is a problem for bull behavior and fertility, especially for non-adapted bulls. As beef farmers usually adopt natural mating as the main reproductive strategy, efficiency in producing calves is usually lower for bulls from non-adapted breeds than that from adapted breeds. However, because of carcass quality, a demand for bulls from European breeds in tropical and subtropical regions still persists.

Gene editing can allow a male to produce sperm from another male, which could be useful for bulls in the tropics. The NANOS homology 2 (NANOS2) belongs to a family of zinc-finger motif-contained RNA-binding protein and it is necessary for generating the spermatogenic cell lineage and for spermatogonial stem cell (SSC) self-renewal (Sada et al., 2009; Shen and Xie, 2010). The CRISPR/Cas9 system has been used to knockout the *NANOS2* gene in pigs to generate male offspring without germline cells (spermatogonia) but with preserved testicular development (Park et al., 2017). The authors have suggested that *NANOS2-*null male offspring may serve as potential surrogates for spermatogonial stem cells transplantation (SSCT). In fact, the phenotype has been replicated and proved feasible in mice, pigs, goats and cattle (Ciccarelli et al., 2020). Adult *NANOS2* knockout surrogate male pigs and bucks have been able to sustain spermatogenesis after SCCT. Moreover, this study confirmed that *NANOS2* knockout male cattle presented a phenotype consistent with germline ablation, expanding the exciting prospect of using the SSCT technique in the cattle industry (Ciccarelli et al., 2020).

**Figure 2:**
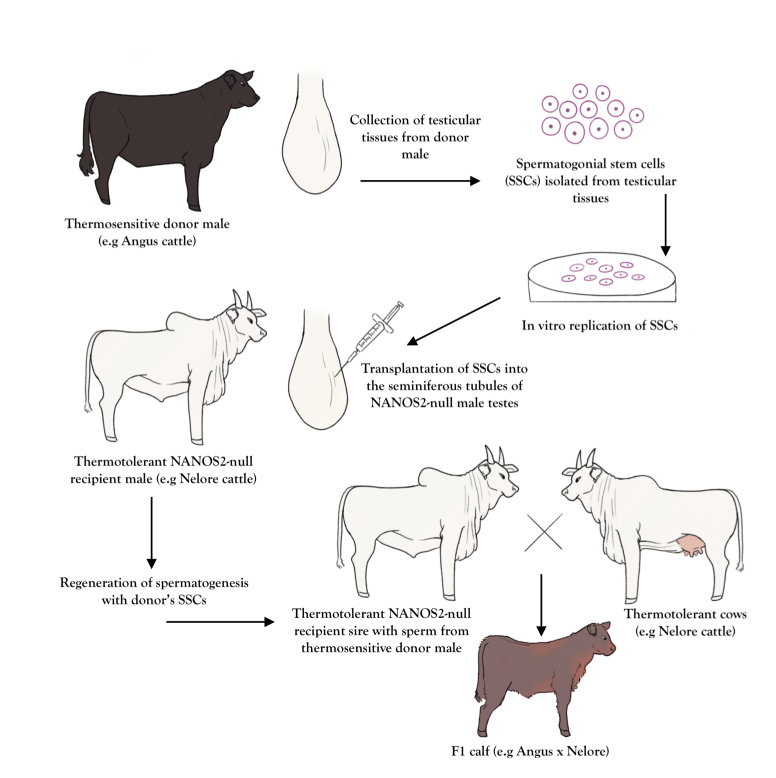
Transplantation of spermatogonial stem cell (SCC) from a thermosensitive breed to the testes of a *NANOS2*-null thermotolerant breed. SCCs from a donor male (e.g Angus cattle) are collected and expanded in vitro before being transplanted into the seminiferous tubules of gene-edited *NANOS2*-null surrogate male (e.g. Nelore cattle). The surrogate males can then be used to breed Nelore cows to produce Angus x Nelore F1 calves in large beef farms in the tropics (adapted from Giassetti et al., 2019).

Applications of gene editing to produce *NANOS2* knockout offspring associated with SSCT may impact positively the field of cattle production. Surrogate sires generated from adapted indigenous breeds (such as zebu breeds) could carry sperm from thermosensitive high genomic value sires (from European breeds) and be used to breed cows by natural mating in the tropical or tropical regions (Figure 2), particularly in regions with low prevalence of artificial insemination use. In animal breeding, genetic gain can be accelerated if spermatogonia are collected from high genomic value male calves at a very young age and transplanted into surrogate knockout adult males, enabling the surrogate male to produce normal sperm from the young donor. Semen from the surrogate sire can then be used for in vitro fertilization of oocytes collected from prepubertal heifers or calves (Baruselli et al., 2021; Silva et al., 2022) thus dramatically reducing generation interval. This latter application can be particularly interesting for breeds with delayed puberty, as found in some indigenous cattle (Cooke et al., 2020).

## Gene editing to modulate cattle methane emissions

One of the gases with high impact on global warming is methane (CH_4_), although it has a short lifetime (Balcombe et al., 2018). In ruminants, during the fermentation process the microbiota in the rumen use H_2_ to reduce carbon dioxide (CO_2_) and produce methane, which is released to the atmosphere mainly through eructation and breathing (Johnson and Johnson, 1995). Cattle is the species that most contribute to methane emissions (Gerber et al., 2013; Black et al., 2021) and countries in tropical and subtropical regions tend to have a greater methane emission (Gerber et al., 2013; Chang et al., 2019). The low productivity plays an important role in the amount of methane emitted, as more animals are required to produce meat and milk in tropical and subtropical regions and, thus, the methane emission per unit of milk or meat produced is high. Indeed, Latin America, Asia and Africa, where cattle have low meat and milk productivity, emit more greenhouse gases and produce less protein from cattle compared with North America and Europe (Gerber et al., 2013). Therefore, increasing productivity and reducing the relative number of animals can be one of the keys to reduce methane emissions in tropical and subtropical zones.

As mentioned in the previous paragraphs, gene editing can be used to increase thermotolerance in European breeds or improve milk yield in indigenous breeds, contributing to generate more productive animals for tropical and subtropical conditions. In that context, the gene editing can indirectly contribute to reduce cattle methane emission per unit of protein produced. Nevertheless, gene editing technologies may potentially be applied to reduce the methane production directly in the rumen. The rumen methane is produced by Archaea and the largest groups in the rumen are *Methanobrevibacter gottschalkii* and *Methanobrevibacter ruminantium* (Henderson et al., 2015). Several enzymes and cofactors are involved in the methanogenic pathway (Shima et al., 2002; Ferry, 2011) and its biochemistry has been widely reported (Ferry, 1992; Deppenmeier, 2002; Ferry, 2011). The genome sequence of *Methanobrevibacter ruminantium* is available (Leahy et al., 2010) as well as the prediction of functional properties of its operome (Bharathi et al., 2020). It has been shown that the genome of Archaea can be manipulated using the CRISPR/Cas system (Li et al., 2016; Nayak and Metcalf, 2017). This knowledge opens the possibilities for the use of gene editing strategies to modulate the methane production in the rumen.

Gene editing with Cas9 has already been used to introduce insertions and deletions via HDR with high efficiency in the archaeon *Methanosarcina acetivorans,* (Nayak and Metcalf, 2017). As most Archaea encode CRISPR/Cas systems, another strategy would be to perform the gene editing using the Archaeon’s own system, requiring only the sgRNA to target a DNA sequence and the ODN donor template for HDR (Li and Peng, 2019). One challenge is to choose the best targets for gene editing, as the wrong targets could generate less competitive methanogens in the rumen microbiome. One option could be to knock down key genes in the methanogenesis pathway. The transcript abundance of genes encoding enzymes involved in the hydrogenotrophic methanogenesis pathway was shown to be lower in the rumen methanogens from sheep with low methane emission compared with those with high emission. The largest differences were found in transcripts from genes that belong to the operon that encodes subunits of methyl-coenzyme M reductase (Shi et al., 2014), important for methane biogenesis. Based on that, one can infer that perhaps the downregulation of genes involved in the hydrogenotrophic methanogenesis can be an adequate strategy to reduce the rumen methane emission.

## Regulatory aspects of gene editing

The approaches used to edit a gene using nucleases can be classified in three categories: site-directed nucleases type 1 (SDN-1), SDN-2 and SDN-3 (Jones, 2015; Sprink et al., 2016). The approach using SDN-1 relies only on NHEJ mechanism (NHEJ) and it can be applied to cause mutations to promote gene knockout or insert a premature stop codon, interfering with protein expression. Because SDN-1 does not insert foreign DNA into the genome, gene-edited organisms generated using this approach can be considered non-genetically modified organisms (GMO) in some countries on a case-by-case analysis. That is the case of Brazil, Argentina, Australia and Japan. On the other hand, the SDN-2 approach relies on a short ODN donor template to repair the DSB by HDR and it can be used to introduce few bases in the genome without introducing foreign DNA, being more precise than SDN-1. Brazil, Argentina and Japan can also consider gene-edited organisms generated by SDN-2 as non-GMO (Whelan and Lema, 2015; Vieira et al., 2021; Jones et al., 2022). In contrast, Australia regulates organisms generated by SDN-2 approach as GMO (Jones et al., 2022).

In the European Union, the Court of Justice decided that products developed by gene editing techniques are subject to the same regulation of GMOs regardless of the approach employed,, although the matter is still under discussion (Tani, 2022). In the United States of America, the Food and Drug Administration (FDA) released a guidance document that proposed to regulate food animals with an intentionally altered genomic (IGA) DNA using molecular technologies as new animal drug (Van Eenennaam et al., 2021); in 2022, however, the FDA performed a risk assessment of SLICK animals generated by gene editing and concluded that the IGA contained in the SLICK cattle posed low risk to people, animals, the food supply and the environment. Thus, there was no objection to introduce the animals or their products in the market and no distinction between facilities to raise conventional animals and gene-edited SLICK cattle was required (U.S. Food and Drug Administration, 2022).

For SDN-3, the DNA repair is also performed by HDR, but usually with a large donor template where an exogenous DNA sequence (a whole foreign gene, part of its sequence or a recombinant DNA) is included. Organisms generated by SDN-3 approach are uniformly considered as GMOs.

Thus, depending on the country, gene-edited products generated by SDN-1 or SDN-2 approaches can be classified as non-GMO on a case-by-case analysis by local regulatory agencies (Table 1). That can be the case of cattle generated with the SLICK, GHR, SCD1 and DGAT1 mutations discussed in this review. Indeed, gene-edited SLICK cattle have already been classified as non-GMO in Brazil and Argentina.

**Table 1:** Classification of gene-edited animals in same countries according to the approaches used to edit the target gene (SDN). Decisions taken by the regulatory agencies to classify gene-edited products as non-GMO are based on case-by-case analysis.

**Country**	**SDN1** **1**	**SDN2^1^**	**SDN3^1^**
Brazil	Non-GMO	Non-GMO	GMO
Argentina	Non-GMO	Non-GMO	GMO
Japan	Non-GMO	Non-GMO	GMO
Australia	Non-GMO	GMO	GMO
European Union	GMO	GMO	GMO
United States of America2	IGA	IGA	IGA

^1^SDN1-3: types of site-directed nucleases approaches (see main text); ^2^Unites States of America: gene-edited animals are classified as intentionally genetically altered (IGA) animals, regardless the SDN approach.

## Final considerations

Cattle farming in tropical and subtropical regions have several challenges imposed by the environment, one of the main ones being heat stress. Heat stress results in low productivity and is one of the main constraints for efficient cattle farming activity in such regions. Gene editing technologies can be applied to decrease the negative effects of heat stress on productivity. Mutations associated with heat tolerance can be inserted in thermosensitive European breeds; similarly, mutations associated with milk yield and composition can be inserted in thermotolerant but low productivity indigenous breeds. Surrogate sires from adapted breeds can carry sperm from non-adapted, high genomic value bulls for natural mating. Increasing cattle productivity in the tropics and subtropics will contribute to produce more animal protein without significantly increasing methane emissions. Finally, gene editing could also be applied to modify the expression of genes in Archaea in order to modulate methane production in the rumen. Those potential applications are summarized in the Table 2. The association of reproductive biotechnologies, gene editing and genomic selection can be applied to generate large numbers of gene edited animals with high estimated genomic value, contributing to boost the genetic improvement and productivity in tropical and subtropical countries.

**Table 2:** Potential applications of genome editing for cattle farming in the tropics.

**Trait**	**Approach**	**Gene**	**Target breeds or specie**
Heat tolerance	Introgression	*PRLR* (alleles Slick)	Holstein/Angus
Milk yield	Introgression	*GHR* (allele Y)	Gir/Girolando
Milk yield and fat	Introgression	*DGAT1* (allele A)	Gir/Girolando
Milk fat	Introgression	*SCD1* (allele A)	Gir/Girolando
Natural mating	Knockout	*NANOS2*	Angus/Nelore
Methane emission	Knockout Down regulation	Methanogenesis genes	Rumen Archaea

Girolando: Holstein x Gir synthetic breed developed in Brazil.
